# Vector competence of the *Aedes aegypti* population from Santiago Island, Cape Verde, to different serotypes of dengue virus

**DOI:** 10.1186/s13071-015-0706-8

**Published:** 2015-02-19

**Authors:** Aires Januário Fernandes da Moura, Maria Alice Varjal de Melo Santos, Claudia Maria Fontes Oliveira, Duschinka Ribeiro Duarte Guedes, Danilo de Carvalho-Leandro, Maria Lidia da Cruz Brito, Hélio Daniel Ribeiro Rocha, Lara Ferrero Gómez, Constância Flávia Junqueira Ayres

**Affiliations:** Departamento de Entomologia, Centro de Pesquisas Aggeu Magalhães (CPqAM), Fundação Oswaldo Cruz- PE, Brasil; Unidade de Ciências da Natureza, da Vida e do Ambiente, Universidade Jean Piaget, Cape Verde; Departamento de Zoologia, Universidade Federal de Pernambuco (UFPE), Programa de Pós-graduação em Biologia Animal, Recife, Brasil

**Keywords:** Dengue, RT-PCR, Vector, NS1 antigen, Cape Verde

## Abstract

**Background:**

Dengue is an arboviral disease caused by dengue virus (DENV), whose main vectors are the mosquitoes *Aedes aegypti* and *Aedes albopictus. A. aegypti* is the only DENV vector in Cape Verde, an African country that suffered its first outbreak of dengue in 2009. However, little is known about the variation in the level of vector competence of this mosquito population to the different DENV serotypes. This study aimed to evaluate the vector competence of *A. aegypti* from the island of Santiago, Cape Verde, to four DENV serotypes and to detect DENV vertical transmission.

**Methods:**

Mosquitoes were fed on blood containing DENV serotypes and were dissected at 7, 14 and 21 days post-infection (dpi) to detect the virus in the midgut, head and salivary glands (SG) using RT-PCR. Additionally, the number of copies of viral RNA present in the SG was determined by qRT-PCR. Furthermore, eggs were collected in the field and adult mosquitoes obtained were analyzed by RT-PCR and the platelia dengue NS1 antigen kit to detect transovarial transmission.

**Results:**

High rates of SG infection were observed for DENV-2 and DENV-3 whereas for DENV-1, viral RNA was only detected in the midgut and head. DENV-4 did not spread to the head or SG, maintaining the infection only in the midgut. The number of viral RNA copies in the SG did not vary significantly between DENV-2 and DENV-3 or among the different periods of incubation and the various titers of DENV tested. With respect to DENV surveillance in mosquitoes obtained from the eggs collected in the field, no samples were positive.

**Conclusion:**

Although no DENV positive samples were collected from the field in 2014, it is important to highlight that the *A. aegypti* population from Santiago Islands exhibited different degrees of susceptibility to DENV serotypes. This population showed a high vector competence for DENV-2 and DENV-3 strains and a low susceptibility to DENV-1 and DENV-4. Viral RNA copies in the SG remained constant for at least 21 dpi, which may enhance the vector capacity of *A. aegypti* and suggests the presence of a mechanism modulating virus replication in the SG.

## Background

Dengue is considered one of the most important arboviral infections in the world. Currently, it is estimated that 3.6 billion people live in tropical and subtropical areas where dengue is endemic. Approximately 50 to 200 million infections occur annually, resulting in 500,000 cases of dengue hemorrhagic fever (DHF) and more than 20,000 deaths [1,2].

Dengue is caused by a flavivirus named Dengue virus (DENV), which is transmitted to humans by mosquitoes of the genus *Aedes*, principally *Aedes aegypti* and *Aedes albopictus* [3]. This virus comprises different serotypes that evolved in nonhuman primates from a common ancestor and entered the urban cycle approximately 100–1500 years ago [4]. Despite the vast knowledge of the virus and the great epidemiological importance of this disease, there are currently no specific antiviral therapies or commercially available vaccines against all serotypes of DENV, which limits the prevention of viral transmission to vector control.

*A. aegypti* is the main vector of DENV in most countries where dengue is endemic [5]. This mosquito originated in Africa but is currently widely distributed across tropical and subtropical regions of Africa, Asia, Australia, the South Pacific, Americas and parts of the Middle East [5,6]. According to Gubler [7], increased geographical distribution of *A. aegypti* has been the main cause of DENV dissemination in the world.

Natural populations of *A. aegypti* exhibit genetic variation that can be responsible for various degrees of susceptibility to dengue virus infection [8-10]. The ability of the vector to be infected by a pathogen, allowing it to replicate and transmit it to another host, is called vector competence [10]. Vector competence is mainly influenced by the genetic variability of the vector and the pathogen; however, it is sometimes modulated by environmental factors [11,12]. Therefore, variation in *A. aegypti* vector competence to DENV observed in different natural environments has implications for the virus’s transmission. Thus, studying this variation is important for understanding the dynamics of DENV transmission in different geographical contexts, and the understanding of the mechanisms that modulate this vector competence can help to develop alternative ways of controlling vector borne diseases, such as genetically modified mosquitoes that are refractory to the virus [12].

Currently, four serotypes of DENV (DENV1-4) co-circulate in Africa, and most epidemics have been caused by DENV-2, followed by DENV-1 [13]. The prevalence of dengue in Africa is low when compared with that in other endemic regions, which according to some authors, may in part be due to the lower vector competence of African strains of *A. aegypti*, especially the subspecies *A. aegypti formosus* [14,15].

The first outbreak of dengue fever in Cape Verde occurred in 2009. Cape Verde is an archipelago located 500 km from the West African coast. During the epidemic, more than 20,000 cases were reported, with 174 diagnosed as DHF, and four people died [16]. The epidemic was caused by a DENV-3 strain, which possibly originated from neighboring countries on the West African coast [17].

The presence of *A. aegypti* in Cape Verde has been recorded since 1931 [18], with no records of other species of *Aedes,* such as *A. albopictus*, that could be implicated in the transmission of DENV [19]. Nevertheless, little is known about the level of vector competence of local populations of *A. aegypti* to DENV. Vazeille et al. [20], demonstrated that the *A. aegypti* population from Santiago Island, Cape Verde, has a moderate ability to transmit the DENV-3 strain responsible for the 2009 epidemic, but the population exhibited little susceptibility to the DENV-2 strain isolated from Thailand.

Considering that all four serotypes are present in the African mainland and that Cape Verde is located in a strategic route connecting Africa, Europe and the Americas, by sea or by air, thus presenting a high risk of introduction of new DENV strains, it is of great interest to know the status of susceptibility to infection of all serotypes in the local *A. aegypti* population in order to implement control measures in advance to prevent future outbreaks.

Therefore, the present study aimed to evaluate the vector competence of *Aedes aegypti* from the island of Santiago, Cape Verde, to four serotypes of dengue virus (DENV- 1, DENV- 2, DENV- 3 and DENV- 4) using RT-PCR and also to survey for DENV serotypes in *Aedes aegypti* egg samples collected from the field. Our results indicate significant variation regarding the mosquito susceptibility to the four serotypes investigated, which has important implications to the epidemiology of dengue in the country.

## Methods

### Study area, sample collection and establishment of the *Aedes aegypti* population

Sample collection was conducted on the island of Santiago, located in the archipelago of Cape Verde. Cape Verde is an African country located in the Atlantic Ocean (17° 12 ′ - 14° 48′ N and 22° 44 ′ - 25° 22′ W), 500 km off the western coast of Africa [21]. It is composed of ten islands (Santo Antão, São Vicente, Santa Luzia, São Nicolau, Sal, Boavista, Maio, Santiago, Fogo and Brava), with a total area of 4,033 km^2^, and has 491,683 inhabitants [22].

Santiago is the largest island with an area of 991 km^2^ and a population of 273,919 inhabitants distributed in nine counties [22]. Santiago is also home to the country’s capital, the city of Praia, which is the most populated city in Cape Verde and where the highest number of dengue cases were reported during the epidemic of 2009 [16].

For the infections experiments, *A. aegypti* eggs were collected using oviposition traps (ovitraps), adapted from the Fay and Perry model [23], from March to June 2012. A total of 107 ovitraps were distributed in peridomestic areas over seven counties of the island close to a variety of potential breeding sites (wells, tanks and other aquatic habitats), according to routine entomological surveys performed by the Ministry of Health of Cape Verde. To search for natural transovarial DENV transmission, eggs were collected during January and February 2014 using a total of 241 ovitraps placed in four sites: Assomada, City of Praia, Santa Cruz and Tarrafal.

The collected eggs were then transported to the insectary of Centro de Pesquisas Aggeu Magalhães in Brazil, where they were hatched according to standard procedures to establish the adult population as described by Carvalho et al. [24] or stored at −80°C in pools of five mosquitoes (separated according sex and site of collection) for the vertical transmission analysis. The insectary conditions were 70 –80% humidity, a 12 h:12 h light:dark cycle, and 26 ± 1°C. Infections experiments described below were performed with the F_1_-F_3_ generations and vertical transmission analysis in the F0 generation.

### Propagation of DENV in the C6/36 cell line

The viral stocks of different DENV serotypes used in this study were generously provided by the Laboratory of Virology and Experimental Therapy (LAVITE) from CPqAM/ Fiocruz-PE, Brazil. The strains used were DENV-1 42735/BR PE (GenBank Accession N° EU259529), DENV-2 3808/BR-PE (EU259569), DENV-3 85469/BR-PE (EU259607) and DENV-4 1385 (U1842). In previous studies, these strains efficiently infected and disseminated in *Aedes aegypti* populations from Recife, Brazil [25,24].

Viral stocks were propagated in the C6/36 cell line, as previously described by Carvalho-Leandro *et al.* [24] and Xi *et al*. [26], with modifications. Briefly, 250 μl of virus stocks was used to infect C6/36 cells (seeded to 80% confluence) with a multiplicity of infection (MOI) of 0.5 virus particles/cell. The cells were then incubated at 28°C for 5–7 days, until strong cytopathic effects were observed. Three cycles of freezing and thawing at −80°C and at 37°C, respectively, were performed to lyse the cells and release the viral particles. Cells and supernatant were then mixed 1:1 with defibrinated sheep blood and used to feed the females. The infected blood was maintained at 37°C for 30 minutes prior to feeding the mosquitoes. For control experiments, a flask of uninfected C6/36 cells (no virus) was maintained under similar conditions and used to create a noninfectious bloodmeal. Before feeding, an aliquot of the blood/virus mixture was stored at −80°C for subsequent virus titration, as described in Santos et al. [27].

### Mosquito infection

Oral infection was performed in nulliparous 7- to 10-day-old female mosquitoes using an artificial membrane feeding system [28]. Approximately 100–150 mosquitoes, starved for 24 hours before the experiment, were fed with blood containing, separately, different DENV serotypes over 45 minutes. Simultaneously, a control group of an equal number of individuals was fed with a noninfectious bloodmeal. After feeding, only engorged females were transferred to another cage and maintained under previously described standard conditions for 21 days. Two independent experiments were performed for each serotype separately.

### Tissue collection and RNA extraction

Heads, midguts and salivary glands were collected on the 7th, 14th and 21st day post- infection (dpi). Dissections were performed in 1X PBS using 15 to 20 mosquitoes per time point. After dissections, the tissues were placed separately in 1.5 ml microtubes containing 250 μl of mosquito diluent (1X PBS, 0.1 M, pH 7.4, 10% fetal bovine serum, 1% antibiotic and antifungal agents) [29] and stored at - 80°C until RNA extraction.

Total RNA extraction was performed using Trizol following the manufacturer’s protocol (Invitrogen Cat. N° 15596–026), and RNAs were further treated with DNase (Turbo DNase - Ambion, Cat N° AM2239) and stored at - 80°C until further use.

### Determination of infection rates by One Step RT-PCR

Detection of the virus in the midgut, salivary glands and head was first performed using One Step RT-PCR and NS5 primers as reported by Kong *et al.* [30]: forward, 5′-GGAAGGAGAAGGACTGCACA-3′ and reverse, 5′-ATTCTTGTGTCCCATCCTGCT-3′.

PCR reactions were performed using 12.5 μl of master mix (Promega Cat.N° M7505), 10 μM of each primer, 0.1 M DTT, 1.05 U of AMV-RT (Invitrogen), 1.2 U of RNAase OUT, 500 ng of the sample RNA, and water to a final volume of 25 μl. Cycling conditions were performed as follows: 1 cycle at 50°C for 60 minutes, 40 cycles at 94°C for 1 minute, 55°C for 1 minute, 72°C for 2 minutes and a final cycle of 72°C for 10 minutes. RT-PCR products were then analyzed by electrophoresis on a 1.5% agarose gel in Tris-borate-EDTA (TBE) buffer with ethidium bromide and visualized on a UV transilluminator.

### Quantification of viral genome in the salivary glands by qRT-PCR

The quantitative RT-PCR (qRT-PCR) was performed using the Quantitect SYBR Green RT-PCR Kit (QIAGEN, Cat N° 204245) and the same primers NS5F and NS5R described above for the One Step RT-PCR.

For the PCR reactions, the following were used: 12.5 μl of SYBR Green Master Mix (2X), 0.2 mM of each primer, 0.25 μl of the reverse transcriptase enzyme (QIAGEN), 5 μl of each RNA sample (normalized to 50 ng) and ultra-pure water to a final volume of 25 μl. Amplification reactions were performed in a PCR real-time system from Applied Biosystems (ABI 7500) programmed for 1 cycle at 50°C for 30 minutes, 95°C for 15 minutes, 40 cycles at 94°C for 15 seconds, 58°C for 30 seconds, and 72°C for 30 seconds. The number of viral RNA copies in the salivary glands was determined by absolute quantification using cycle threshold (ct) values from a standard curve method included in each PCR plate. The standard curve consisted of known concentration of purified NS5 transcript as described in Kong *et al*. [30].

### Determination of viral titer in the mixture blood/virus by plaque assay in C6/36 cells

Virus titration was determined by a focus-forming assay in C6/36 cells, as described previously by Santos *et al.* [27]. Briefly, 3 x 10^5^ C6/36 cells in the L15 medium (supplemented with 5% fetal bovine serum) were seeded in a 24-well plate 48 hours before the test. Subsequently, the cells were incubated with serially diluted virus-containing blood for 1 hour. The L-15 medium was removed, and the cells were covered with 1 ml of semisolid medium containing carboxymethylcellulose (1%), L-15 medium (2X concentrate), FBS (2%), and antibiotic and antifungal agents (1%) and incubated at 28°C for 5 days. After the incubation period, the cells were fixed with 30% cold acetone in 1X PBS for 13 minutes and rinsed once with PBS and allowed to dry for 24 hours. After this period, the cells were incubated for 1 hour at 37°C with the primary antibody (HMAF diluted 1:100 in ligation buffer). The plates were washed three times with wash buffer (1X PBS and Tween-20) and incubated with horseradish peroxidase-conjugated recombinant protein G (Invitrogen- Cat No. 10–1223) at 37°C for 1 hour. The plates were rinsed three times with wash buffer; finally, 500 μl of AEC substrate (Sigma- Cat. N° A6926) was added, and the plate was incubated until the appearance of foci. Foci were counted, and virus titers are expressed in focus-forming unit/ml (FFU/ml).

### Data analysis

The midgut infection rate, dissemination rate and vector competence (salivary glands infection rate) were determined for each DENV serotype. The midgut infection rate corresponded to the number of mosquitoes with infected midgut divided by the total number of mosquitoes exposed to the virus. The dissemination rate was determined by the number of mosquitoes with infected head divided by the total number of mosquitoes with infected midgut. The vector competence or salivary glands (SG) infection rate was defined as the number of mosquitoes with infected salivary glands divided by the total number of mosquitoes exposed [10].

ANOVA with Dunnett’s post-test and nonparametric Kruskal Wallis and Mann–Whitney tests were applied for the comparative analysis, using SigmaPlot Version 12.5 software.

### Dengue virus detection through RT-PCR and Platelia Dengue NS1 Ag kit

Eggs were collected in four sites of the island of Santiago (Assomada, Praia, Santa Cruz and Tarrafal) and virus detection in adult mosquitoes was performed via RT-PCR and the platelia dengue NS1 kit. Mosquito pools (up to 5 specimens) were homogenized with a pestle in 500 μl of Leibovitz medium (L-15) supplemented with 2% Fetal Bovine Serum (FBS), Penicillin-Streptomycin (1%) and Fungizon (1%). For RT-PCR assays, viral RNA was extracted from mosquito samples using Trizol according to the manufacturer’s protocol. Prior to RT-PCR reactions, the RNAs were treated with TURBO™ DNAse (Ambion) to avoid genomic DNA contamination. RT-PCR for detecting and genotyping DENV in mosquito pools was performed according to Lanciotti *et al.* [31]. For Platelia Dengue NS1 Ag. (BioRad) assay, fifty microliters (50 μl) of the supernatant of centrifuged mosquito homogenate suspensions was used. Tests were performed as described in the manufacturer’s protocol.

## Results

### Infection with DENV-1

DENV-1 was detected in midgut only at 21 dpi, reaching a midgut infection rate of 13% in the 1st experiment (viral titer of 5 × 10^4^ FFU/ml) and 27% in the 2nd experiment (2 × 10^5^ FFU/ml) (Table 1).

**Table 1:** **Midgut infection rate, dissemination rate and vector competence estimated by RT-PCR using NS5 gene for the** ***Aedes aegypti*** **population from Santiago Island, Cape Verde**

**Serotype**	**Virus titer (FFU/mL)**		**Midgut infection rate (%)** ^**a**^						**Dissemination rate (%)** ^**b**^						**Vectorial competence (%)** ^**c**^					
	**1st exp.**	**2nd exp.**	**1st exp.**			**2nd exp.**			**1st exp.**			**2nd exp.**			**1st exp.**			**2nd exp.**		
			**7 dpi**	**14 dpi**	**21 dpi**	**7 dpi**	**14 dpi**	**21 dpi**	**7 dpi**	**14 dpi**	**21 dpi**	**7 dpi**	**14 dpi**	**21 dpi**	**7 dpi**	**14 dpi**	**21 dpi**	**7 dpi**	**14 dpi**	**21 dpi**
DENV-1	5.0 × 10^4^	2 × 10^5^	0.0	0.0	13.3	0.0	0.0	26.7	-	-	100	-	-	25	0.0	0.0	0.0	0.0	0.0	0.0
DENV-2	1.4 × 10^5^	2 × 10^5^	50.0	75.0	75.0	70.0	85.0	60.0	20.0	73.3	93.3	28.6	76.5	91.7	5.0	55.0	65.0	5.0	65.0	35.0
DENV-3	1.0 × 10^6^	2 × 10^6^	10.0	65.0	70.0	15.0	65.0	80.0	50.0	76.9	121.4	66.7	76.9	87.5	5.0	50.0	75.0	0.0	45.0	55.0
DENV-4	1.0 × 10^6^	2 × 10^6^	0.0	0.0	9.0	0.0	0.0	0.0	-	-	0.0	-	-	-	0.0	0.0	0.0	0.0	0.0	0.0

Notes: ^a^Midgut infection rate corresponds to the number of mosquitoes with infected midgut divided by the total number of mosquitoes exposed to the virus. ^b^Dissemination rate corresponds to the number of mosquitoes with infected head divided by the total number of mosquitoes with infected midgut. ^c^The vector competence or salivary glands infection rate corresponds to the number of mosquitoes with infected salivary gland divided by the total number of mosquitoes exposed.

DENV-1 was detected in the mosquito head at 21 dpi as well, with a dissemination rate of 100% in the 1st experiment (5 × 10^4^ FFU/ml) and 25% in the 2nd experiment (2 × 10^5^ FFU /ml). Analyses of the salivary glands (SG) revealed that for both experiments (different titers) these organs did not become infected with DENV-1 during an extrinsic incubation period of 21 days.

### Infection with DENV-2

Unlike DENV-1, DENV-2 was detected in the midgut at 7 dpi, reaching infection rates of 50% and 70% in the 1st (1.4 × 10^5^ FFU/ml) and 2nd experiment (2 × 10^5^ FFU/ml), respectively. After 7 dpi, there was an increase in the midgut infection rate, reaching 75% and 85% at 14 dpi. At 21 dpi, rates of 75% and 60% were recorded (Table 1).

With respect to the DENV-2 dissemination rates, there was an increase in the number of positive head samples during the extrinsic incubation period, reaching a maximum of 93.3% and 91.7% at 21 dpi for the 1st (1.4 × 10^5^ FFU/ ml) and 2nd (2 × 10^5^ FFU/ml) experiments, respectively (Table 1).

At 7 dpi, the percentages of infected salivary glands (vector competence) in both experiments were 5%. For the 1^st^experiment, the maximum was 65% at 21 dpi, and for the second one, it was also 65% but was reached earlier, at 14 dpi.

### Infection with DENV-3

Oral infections with DENV-3 were performed with a virus titer of 1 × 10^6^ FFU/ml (1st experiment) and 2 × 10^6^ FFU/ml (2nd experiment). At 7 dpi, the midgut infection rates were 10% and 15%, which increased with the extrinsic incubation period, reaching 70% (for 1 × 10^6^ FFU/ml) and 80% (2 × 10^6^ FFU/ml) at 21 dpi (Table 1). Dissemination rates of DENV-3 also increased with the extrinsic incubation period, reaching a maximum of 121% and 88% for the 1st and 2nd experiment, respectively.

Similarly to DENV-2, positive SG were detected at 7 dpi, but only in the 1st experiment. The maximum rate observed was 75% for the 1st experiment (1 × 10^6^ FFU/ml) and 55% for the 2nd experiment (2 × 10^6^ FFU/ml).

### Infection with DENV-4

DENV-4 titers in the bloodmeal were 1 × 10^6^ and 2 × 10^6^ FFU/ml (1st and 2nd experiment, respectively).

DENV-4 was only detected in the experiment with the lowest viral titer (1 x 10^6^ FFU/ml), with a midgut infection rate of 9% at 21 dpi. For both experiments, DENV-4 was not detected in the head or the SG of the mosquitoes (Table 1).

### Quantification of viral RNA in the salivary glands

**Figure 1:**
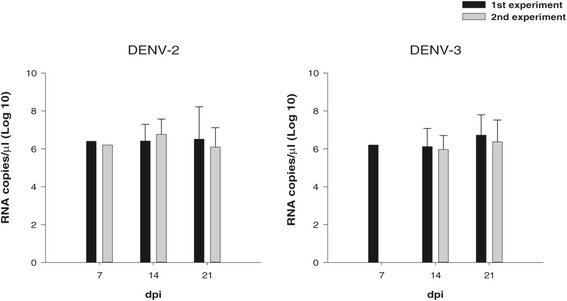
**Quantification of viral RNA copies (DENV-2 and DENV-3) by qRT-PCR detected in the salivary glands of** ***Aedes aegypti*** **collected in Santiago Island, Cape Verde.**

With respect to DENV-2, the number of viral RNA copies in the salivary glands ranged from 2.5 × 10^6^ to 1.8 ×10^8^ in the 1st experiment and from 1.7 × 10^6^ to 3.0 × 10^7^ RNA copies/μl in the 2nd experiment (Figure 1). No significant differences were recorded in the number of copies among the different time points of the 1st experiment (P = 0.9, P > 0.05) nor in the 2nd experiment (P = 0.1, P > 0.05). Comparison between the two experiments also revealed no significant difference (p > 0.05).

DENV-3 RNA copies ranged from 1.7 × 10^6^ to 4.0 × 10^7^ in the 1st experiment. For the 2nd experiment, DENV-3 RNA copies ranged from 5.5 × 10^6^ to 5.2 × 10^7^ RNA copies/μl (Figure 1). Likewise, for DENV-2, no significant differences were observed in the number of copies among the different time points in the 1st experiment (P = 0.16, P > 0.05), nor in the 2nd experiment (P = 0.15, P > 0.05). Furthermore, no differences between the two experiments were detected (P > 0.05).

Comparison of the number of copies of viral RNA from DENV-2 and DENV-3 showed no significant difference between the two serotypes (P > 0.05).

### DENV surveillance in *A. aegypti* mosquitoes collected from field-caught eggs

A total of 6,038 eggs were collected in the four sites; 832 eggs from Assomada, 1,825 from the city of Praia, 2,223 from Santa Cruz and 1,158 eggs from Tarrafal. A total of 1,246 mosquito specimens (252 pools) comprising 533 females (108 *pools*) and 713 males (144 *pools*) were analyzed by RT-PCR and for DENV NS1 antigen using the Platelia Dengue NS1 Ag kit. All pools were negative for both techniques.

## Discussion

This study represents the first evaluation of the vector competence of the *Aedes aegypti* population from Santiago Island, Cape Verde, to four serotypes of Dengue Virus (DENV-1, DENV-2, DENV-3 and DENV-4). According to the results, the *Aedes aegypti* population has high vector competence for DENV-2 (3808/BR-PE) and DENV-3 (85469/BR-PE) but has low susceptibility to DENV-1 (42735/BR-PE) and DENV-4 (1385).

The DENV-1 strain used in this study was isolated in Pernambuco State, Brazil, in 1997, and belongs to the America/Africa genotype [32]. This genotype includes strains isolated in the Americas, India and Africa that have large epidemiological impact throughout the world [33-35]. The transmission of DENV-1 is also common on the African continent [13]. Until mid-2013, more than 517 cases of DENV-1 infections were detected in Angola. Viral genome sequencing revealed that the DENV strain belongs to the America/Africa genotype and is closely related to strains circulating in the Ivory Coast, Abidjan and South America [36]. Additionally, samples collected from European travelers from 2000 to 2008 showed that strains belonging to the America/Africa genotype are circulating in the Ivory Coast, Sudan and Cameroon, thus indicating the epidemiological importance of this genotype in Africa [37].

Despite the importance of this genotype in other countries, our results showed that the replication of DENV-1 in the *A. aegypti* population from Santiago Island was very low, once we were not able to detect virus particles in the salivary glands (SG), even in mosquitoes with infected heads, possibly indicating the presence of an effective SG infection barrier against DENV-1. A previous infection study using an *A. aegypti* population from Recife (Brazil) and the same DENV-1 strain used in our study reported that the infection of the SG in that population occurred at 7 dpi [25], which highlights a large difference in vector competence between the population of *A. aegypti* from Santiago Island and Recife, Brazil. This behavior may help to explain the fact that DENV-1 has never been isolated in Cape Verde, although this serotype continues to circulate in many African countries.

In contrast to what we observed for DENV-1, the population from Santiago Island showed a high vector competence for the DENV-2 serotype. According to Salazar et al. [28], a short period of extrinsic incubation may have important consequences on dengue transmission because the more quickly the virus reaches the SG, the more likely it is to be transmitted to a host during the vector survival period. DENV-2 used in the present study is representative of the Southeast Asian genotype [32]. DENV-2 was first detected in the Americas during an epidemic in Cuba in 1981, when thousands of cases of dengue hemorrhagic fever were documented [38]. Subsequent outbreaks of DHF emerged in Brazil, Venezuela, Colombia, and Guyana [39]. Given that the population of *A. aegypti* from Santiago Island displayed a high vector competence for Southeast Asian DENV-2, immediate preventive measures should be taken if this genotype is detected in Cape Verde or neighboring countries.

Similarly to what was observed for DENV-2, the *A. aegypti* population from Santiago exhibited high vector competence for the DENV-3 strain. This strain was also isolated in Pernambuco, and phylogenetic studies revealed that the strain belongs to the sub-continental India genotype, according to the classification of Rico-Hesse [32], or genotype III, according to Chen and Vasilakis’ classification [40]. In Africa, DENV-3 was first isolated in 1984 during an epidemic of dengue fever in Mozambique [41]. Ten years later, this serotype was detected in Somalia and near the Persian Gulf [42] and then expanded to the West African areas, being isolated in Cameroon (2006), the Ivory Coast (2008), Senegal and Cape Verde in 2009 [17].

The high rates of SG infection by DENV-3 observed in this study can help us to understand the outbreak reported in 2009 in Cape Verde; however, this genotype was not subjected to phylogenetic studies, and therefore is unknown. Vazeille et al. [20] evaluated the vector competence of *A. aegypti* from Santiago Island for a DENV-3 strain isolated in the epidemic of 2009, and the authors observed rates of dissemination of 80% at 14 dpi. However, analysis of saliva by immunofluorescence revealed a transmission rate of only 20%, which, according to the authors, may indicate the presence of an SG infection barrier. The use of immunofluorescence instead of qRT-PCR and saliva in place of SG in the Vazeille et al. study may explain the differences found relative to the present study. In summary, both studies corroborated that the *A. aegypti* population from Cape Verde is efficient in transmitting DENV-3 despite differences in the transmission rate. So far, all strains of DENV-3 detected in East Africa from 1984 to 1993 and also detected in recent dengue cases in Cameroon, Ivory Coast and Senegal (West Africa) belonged to genotype III [17,43], this strain may be the genotype that circulated in Cape Verde at the time of the epidemic.

With respect to DENV-4, the virus replicated less than the three other serotypes analyzed. This result may suggest that the population of *A. aegypti* from Santiago Island has an effective midgut escape barrier (MEB) against DENV-4, and therefore, the virus could not disseminate to SG.

The DENV-4 strain used in this study is representative of genotype II [44], being directly associated with strains of great importance in the Caribbean and South America [45]. Our results indicate that despite the great dispersal ability of the DENV-4 genotype II and the intense flow of people between Santiago Island and Brazil, the island seems to be at a very low risk of its transmission, due to the very low susceptibility of the local mosquito population to this genotype.

With regard to the different virus titers used in oral infections, the titers mainly affected the midgut infection rate because the higher DENV titers used for DENV-1, DENV-2 and DENV-3 resulted in higher rates of midgut infection. Bennett *et al*. [10] demonstrated that the virus titer greatly affects the midgut infection rate. However, the authors found no relationship between the titer and the rate of dissemination. In other experiments, Bosio *et al*. [46] also found no correlation between the titer of DENV- 2 in the midgut and the rate of dissemination. Our results demonstrate that for some viral titers, virus replication in the head increases, whereas replication in the midgut decreases throughout the extrinsic incubation period. This relationship also proved to be important for vector competence because in the 1st experiment, SG infection rate was higher (75%) compared with the 2nd experiment (55%). Salazar et al. [28] also found that DENV-2 replication in the midgut decreases after 10 dpi, whereas the number of infected heads increased after 14 dpi.

Interestingly, viral RNA quantification results in the SG revealed that the amount of RNA stayed relatively constant after reaching these organs and remained constant until the last time point analyzed (21 dpi). This finding demonstrates that the mosquito maintains its ability to transmit the pathogen for a period of at least 21 days. Our results also corroborate those of a previous study where viral RNA quantification in three populations of *A. aegypti* from the state of Pernambuco exhibited no significant differences in viral load in the SG among those populations [24]. According to Salazar *et al.* [28], a relatively constant number of RNA copies could also suggest the existence of some mechanism that regulates viral replication in the mosquito.

Despite many studies focusing on the vector competence of *Aedes* mosquitoes to DENV, the mechanisms that regulate this trait are not fully understood. Vector competence appears to be associated with certain barriers to infection, including the midgut infection barrier (MIB), the midgut escape barrier (MEB) and the SG infection barrier (SIB) [47]. According to Black *et al*. [48], the MIB inhibit the early stages of viral infection, such as binding to receptors, uncoating, transcription and translation, whereas the MEB may act at the level of the passage through the basal lamina or maturation of the virus.

Vector competence can also be modulated by environmental factors. Carrington et al. [49] observed that large temperature fluctuations during the day reduced the rate of infection of the intestine and increased the period of extrinsic incubation of DENV-1 in the mosquito *A. aegypti*. Ramirez *et al*. [50] found that *A. aegypti* mosquitoes infected with certain bacterial isolates from the field were less susceptible to DENV infection.

In Africa, some mosquito populations have shown a low vector competence to DENV, and according to some authors, this low competence seems to somehow explain the lower incidence of dengue on this continent; it is also possible that cases have been underestimated, due to the absence of diagnostic tools in the healthcare setting on this continent [51].

Although the natural transovarial dengue virus transmission may not be a rare event [52-54] and although the dengue transmission in the island of Santiago may still be active on a small scale, there were no positive pools for the eggs collected in the field in this study, suggesting that this is a very rare phenomenon in Cape Verde. With respect to the low vector competence hypothesis, African strains of *A. aegypti*, especially the subspecies *A. aegypti formosus* and the species *A. albopictus,* exhibited lower susceptibility to all four serotypes of DENV [14,15]. Bosio, Beaty and Black [46] observed a higher midgut infection barrier (MIB) in *A. aegypti formosus* when compared with the subspecies *A. aegypti aegypti*. According to the authors, there is evidence that the frequency of the alleles that control the MIB differ greatly between the two subspecies. However, further studies are urgently needed to better understand the transmission of DENV in Africa.

## Conclusion

Although we could detect no DENV circulating in the population of *A. aegypti* from Santiago Island, Cape Verde, during an entomological surveillance in early 2014, our results indicate that this mosquito population exhibits high vector competence for the DENV-2 and DENV-3 strains and appears to be less susceptible to DENV-1 and DENV-4. Viral RNA copies in the SG remained relatively constant for at least 21 days after infection, which may enhance the vector capacity of *A. aegypti*. The results also suggest the presence of some type of modulation mechanism of virus replication in the salivary tissue.
